# Prime and boost aerosol exposure via fog machine or shisha smoke followed by cinnamon hypersensitivity and anaphylaxis to spiced food

**DOI:** 10.1186/s40413-016-0091-6

**Published:** 2016-01-27

**Authors:** Erika Jensen-Jarolim, Franziska Roth-Walter, Erich Leitner, Stefan Buchleitner, Harald Vogelsang, Tamar Kinaciyan

**Affiliations:** Institute of Pathophysiology and Allergy Research, Center of Pathophysiology, Infectiology and Immunology, Medical University Vienna, Vienna, Austria; AllergyCare, Allergy Diagnosis and Study Center, Vienna, Austria; Comparative Medicine, The Interuniversity Messerli Research Institute, Univ. of Veterinary Medicine Vienna, Medical University Vienna and University Vienna, Vienna, Austria; Institute of Analytical Chemistry and Food Chemistry, Graz University of Technology, Graz, Austria; Divison of Gastroenterology, Univ. Clinic of Internal Medicine III, Medical University Vienna, Vienna, Austria; Division of Immunology, Allergy and Infectious Diseases, Department of Dermatology, Medical University Vienna, Währinger G. 18-20, 1090 Vienna, Austria

**Keywords:** Fragrance allergy, Food allergy, Cinnamon aldehyde, Cinnamaldehyde, Type I allergy, Fog machine, Smoke, Shisha, Anaphylaxis, Adverse reaction, Cola

## Abstract

**Background:**

Cinnamon aldehyde (alias cinnamaldehyde) is widely used in food, textile or cosmetic industry. It is mostly associated with contact allergy, but immediate type allergies have been reported. The present study was triggered by a case of anaphylactic events to cinnamon in food and upon skin prick test. We investigated a possible correlation of exposure to a disco fog machine and/or shisha consumption with immediate type hypersensitivity to cinnamon aldehyde in the patient and healthy volunteers.

**Methods & Results:**

In both fog machines and shisha pipes heating of glycerol-based fluids before evaporation renders chemical transversion to malodorous acrolein. Therefore, both methods are frequently operated with aroma additives. Cinnamon aldehyde and derivatives could be detected by gas chromatography in sampled fog flavored with cola fragrance. The patient as well as healthy (mostly female) volunteers were skin prick tested using cinnamon aldehyde diluted in 0.9 % NaCl, Vaseline® or fog fluid. Persons with a history of exposure to disco fog or shisha (*n* = 10, mean 32.8 years) reacted with a significantly larger wheal and flare reaction in the skin test (*p* = 0.0115, *p* = 0.0146, or *p* = 0.098) than the non-exposed (*n* = 8, mean 37.3 years). Both groups were gender matched, but differed in the mean age by 4.5 years. This reaction was specific as compared to skin reactivity to cinnamon alcohol, with only a trend to higher reactivity in exposed persons (ns).

**Conclusion:**

From our data we conclude that hapten fragrances such as cinnamon aldehyde may during heating in glycerol fluids associate to complete antigens and via inspiration lead to specific immediate type hypersensitivity. In some cases the hypersensitivity may be unmasked by spiced food containing cinnamon aldehyde or related chemicals, and lead to severe adverse reactions.

**Electronic supplementary material:**

The online version of this article (doi:10.1186/s40413-016-0091-6) contains supplementary material, which is available to authorized users.

## Background

Cinnamon is broadly used in cosmetics, room deodorants, and as flavor in food. In particular, the non-protein cinnamon compounds like cinnamon aldehyde (cinnamaldehyde), cinnamon alcohol and cinnamic acid are cross-sensitizing contact allergens with a strong percutaneous sensitization potency [1, 2]. In a recent update on sensitization rates in 4200 patients cinnamon aldehyde was among the six allergens with statistically increasing impact [3]. They are all benzaldehyde derivatives (Additional file 1: Figure S1A) and responsible for the antibacterial, immunosuppressive [4] and anti-inflammatory characteristics [5, 6] of cinnamon. Oral contact allergy to cinnamon aldehyde has been associated with the consumption of chewing gums [7, 8], mouthwash, candy, toothpaste [8–10], and diet cola [11]. Oral contact allergy to cinnamon has sometimes been mistaken as angioedema [12]. Also orofacial granulomatoses potentially have a contact-allergic origin and have accordingly been successfully treated with cinnamon- and benzoate free diet [13]. Also airborne sensitization may initiate delayed type allergy to cinnamon and cross-sensitization to other fragrances, especially in occupational settings [14]. Occupational allergy to cinnamon has been observed in bakers [15], in chefs, kitchen or restaurant workers [16], and in physiotherapists [17]. To enhance consumers’ safety EU regulations foresee the labeling of fragrances on products. In a study hexyl cinnamal with 42 % of cases was among the top six most frequently labelled fragrances. 11 % of investigated products were labeled “aroma” or “perfume”, which is problematic for fragrance hypersensitive persons.

Rarely immediate type hypersensitivity with positive skin tests and specific IgE were described in spice factory workers [18]. This is remarkable as in a study 87,5 % of the investigated workers after 5 years of exposure suffered from immediate type and irritant symptoms, 22 % from asthma, whereas none of them had contact dermatitis [19]. It has been discussed whether the cinnamon derivatives may play a role in immediate type allergy [20]. Cinnamon has been described to cause urticaria [21] and there is sporadic evidence that sensitization to fragrances, including alpha-hexyl cinnamon aldehyde, cinnamic alcohol, eugenol, amyl cinnamic aldehyde, and others, can lead to specific gastrointestinal symptoms and angioedema 2 h after ingestion [22].

Taken together, the potential role of cinnamon derivatives in immediate type allergy is still discussed [20]. This is on the one hand due to the irritant aspect of cinnamon and other spices that directly affect the epithelial barrier [23] and thereby complicate the diagnosis, for instance by patch testing [24]. On the other hand, it is insufficiently understood how small chemical compounds could form complete antigens able to sensitize in the immunologic sense. Prompted by a clinical observation of immediate type reaction to cinnamon, we aimed to dissect circumstances under which cinnamon could cause allergic sensitization. An association to cinnamon sensitization was found in the use of fog machines or shisha pipes, in which cinnamon fragrances are often used und may upon heating with glycerol derivatives assemble to complete allergens capable of respiratory sensitization.

## Results

### A patient experiencing severe adverse reactions to spiced food after being exposed to a fog machine and shisha smoke

**Fig. 1:**
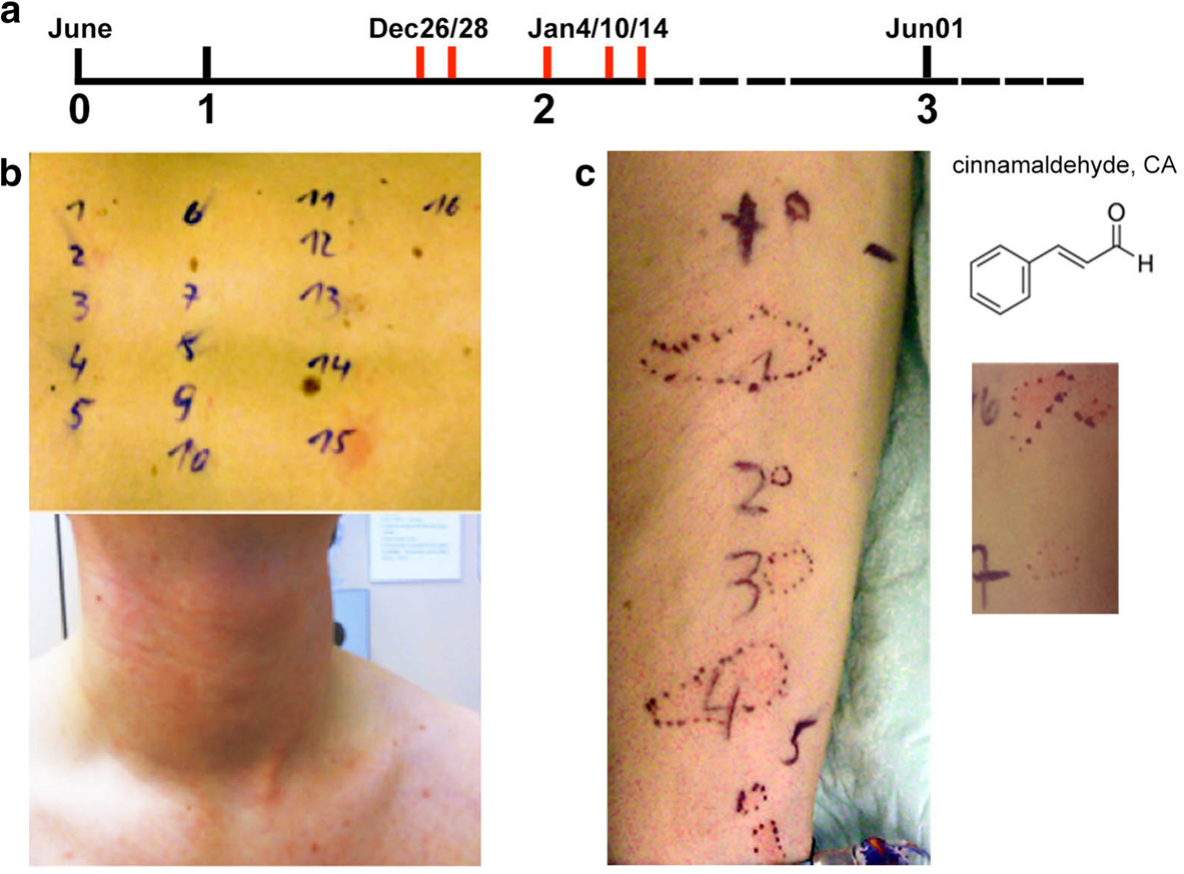
Timeline of case history and clinical pictures of the patient with cinnamon anaphylaxis. Panel **a** Time line: (1) purchase of fog machine; (2) first skin test diagnosis (clinical pictures panel **b**); (3) 2nd skin test (panel **c**). Red bars: anaphylactic events. Panel **b** Prick-to-prick test with suspected food components 1-16 (cinnamon = 15) elicited flush and anaphylaxis 15 min later; panel **c**) 2nd skin prick test with 1: 3 % cinnamaldehyde (CA); 2: 3 % CA in glycerol; 3: 3 % CA in glycerol, boiled; 4: 3 % CA in smoke fluid Eurolite®; 5: 3 % CA in smoke fluid Eurolite®, boiled; 6: 1 % CA in Vaseline®; 7: 1 % Cinnamon alcohol in Vaseline®

A 19 years old young man, non-atopic and without history of allergies or any other diseases, reacted repeatedly with severe adverse episodes to flavored food as well as upon diagnostic prick-to-prick test (Fig. 1a). Whereas the first reaction was associated with cinnamon consumption, trigger of events 4 and 5 were rosemary or mustard. Six months before the first event the patient had purchased a professional dance floor-fog machine. He used the machine at 3-week intervals in several home parties adding cola fragrance to the smoke fluid, and did in the beginning not notice any adverse effects. Besides, the patient was also sporadically exposed to shisha smoke, supplemented with the cinnamon-containing fragrance “double apple”.

**The first anaphylactic episode** happened 2 h after a cinnamon-poppy parfait, a possible time frame in type I food allergy.[25] The patient developed nausea, pharyngeal edema, flush, cold-sweaty hands and anxiety. The patient was treated in an emergency unit with intravenous antihistamines and steroids.

The **second food-induced reaction** occurred 2 days later after a vanilla ice dessert containing almonds and cinnamon. The reaction could be controlled by immediate oral antihistamines and horizontal bedding.

**The third event** occurred under the diagnostic work-up in the allergy clinic with peripherally inserted intravenous access. Among standard skin prick allergens and 16 foods that were prick-to-prick tested, the patient reacted singularly to cinnamon with a wheal and flare reaction (Fig. 1b). 15 min later he developed flush, globus sensation and cold sweaty hands. The situation could be controlled by antihistamines and steroids i.v.

**The fourth and fifth events** were experienced due to rosemary-spiced chicken (flavor p-cymene) and then to mustard (flavor substance 4-hydroxy-benzyl-isothiocyanate) (Additional file 1: Figure S1A). Reaction 4 could be controlled by a military medic, reaction 5 by the patients’ family, with oral antihistamines and horizontal bedding.

The patient was prescribed rescue medication including anti-histamine, oral corticosteroids and EpiPen® (epinephrine) autoinjector. The differential diagnoses of systemic mastocytosis and neuroendocrine tumors were ruled out. ImmunoCAP-FEIA (Phadia, ThermoFisher, Stockholm, Sweden) test for specific IgE to cinnamon and vanilla remained negative, suggesting that sensitization was possibly not directed to protein compounds. Gastroenterological examinations showed slight esophageal erosions and enhanced duodenal permeability in the sucrose-lactose-mannose test as described for food allergies.[26]

To evaluate the status of sensitization 5 months later, the patient was skin prick tested using peripherally inserted intravenous access. Due to the severeness of the sensitization oral food provocation was not attempted and also refused by the patient. To this time point he showed positive immediate type skin test reactions to 3 % cinnamon aldehyde or -alcohol in various aqueous or fatty formulations: in glycerol, glycerol boiled, in smoke fluid Eurolite®, in smoke fluid Eurolite® boiled, or to 1 % CA or 1 % cinnamon alcohol in Vaseline® (Fig. 1c). Boiling the fluids before testing for 5 min at 95 °C did not change the reaction. The results indicated persistence of the specific cinnamon hypersensitivity in the patient. Putting the patient on a flavor-free, salt-only diet since prevented further reactions.

### Analysis of evaporations derived from a fog machine

Smoke fluids contain 85 % higher alcohols (polyethylene glycol, glycerol, triethylene glycol, 1,2-propylene glycol; Additional file 1: Figure S1B) which render droplets in the micrometer range when sprayed at 180–290 °C. During heating glycerol polymerizes and transforms into related chemicals, among them acrolein (Additional file 1: Figure S1B, C). Fog machine users camouflage the bad smell of acrolein with fragrances, among them cola flavour being especially preferred. When smoking the shisha pipe a similar principle takes place. Tobacco leaves are prepared in glycerol fluid to enhance their elasticity. As during hot evaporation malodorous acrolein is formed, shisha consumers usually add fragrances, such as “Double apple” typically containing cinnamon fragrance. Smoke samples from a professional smoke machine driven with glycerol fluid alone, or with glycerol fluid containing cinnamon aldehyde, or the commercial fragrance “cola” were collected after 15 and 30 min using a portable field sampler, and analyzed by gas chromatography.

In the 15 min samples of smokes supplemented with cola fluid, besides higher alcohols, cinnamon aldehyde could be detected as a predominant substance (Table 1). In the 60 min samples the same major compounds showed the typical instability and chemical transversion of glycerol products.

**Table 1:** Chemical compounds detected in collected fog fluids by chromatography, operating fog machine for 15 or 60 min

*time point*	*abundance*	*compound*	*time*	*abundance*	*compound*
Nebulizer fluid alone:					
15 min			60 min		
9,168	393.059	1,3,5-Trioxane			
10,156	8.957.277	1,2-Propanediol	10,255	15.897.840	1,2-Propanediol
14,244	90.466.244	Diethylene glycol*)	14,382	141.211.849	**Diethylene glycol**
17,624	821.030	Triethylene glycol	17,658	1.945.857	Triethylene glycol
Nebulizer fluid + Cola fragrance:					
15 min			60 min		
10,067	2.239.495	1,2-Propanediol	10,061	1.943.051	1,2-Propanediol
14,097	48.148.062	**Diethylene glycol**	14,132	54.836.129	**Diethylene glycol**
14,997	712.992	p-Cymol			
15,088	2.295.324	dl-Limonene			
17,408	2.350.828	beta-Fenchyl alcohol	17,411	1.056.367	beta-Fenchyl alcohol
18,445	14.854.717	**Cinnamaldehyde**	18,482	53.236.624	**Cinnamaldehyde**
			21,035	299.127	beta-Bisabolene
Nebulizer fluid + Cinnamaldehyde:					
15 min			60 min		
			10,071	2.714.874	1,2-Propanediol
14,041	48.061.979	**Diethylene glycol**	14,178	55.795.838	**Diethylene glycol**
17,605	597.330	Triethylene glycol			
17,747	574.291	Cinnamaldehyde	17,748	2.220.382	Triethylene glycol
18,497	98.256.850	**Cinnamaldehyde**	18,551	324.124.086	**Cinnamaldehyde**

*) Most abundant substances indicated in bold

### Skin prick testing in healthy volunteers

**Fig. 2:**
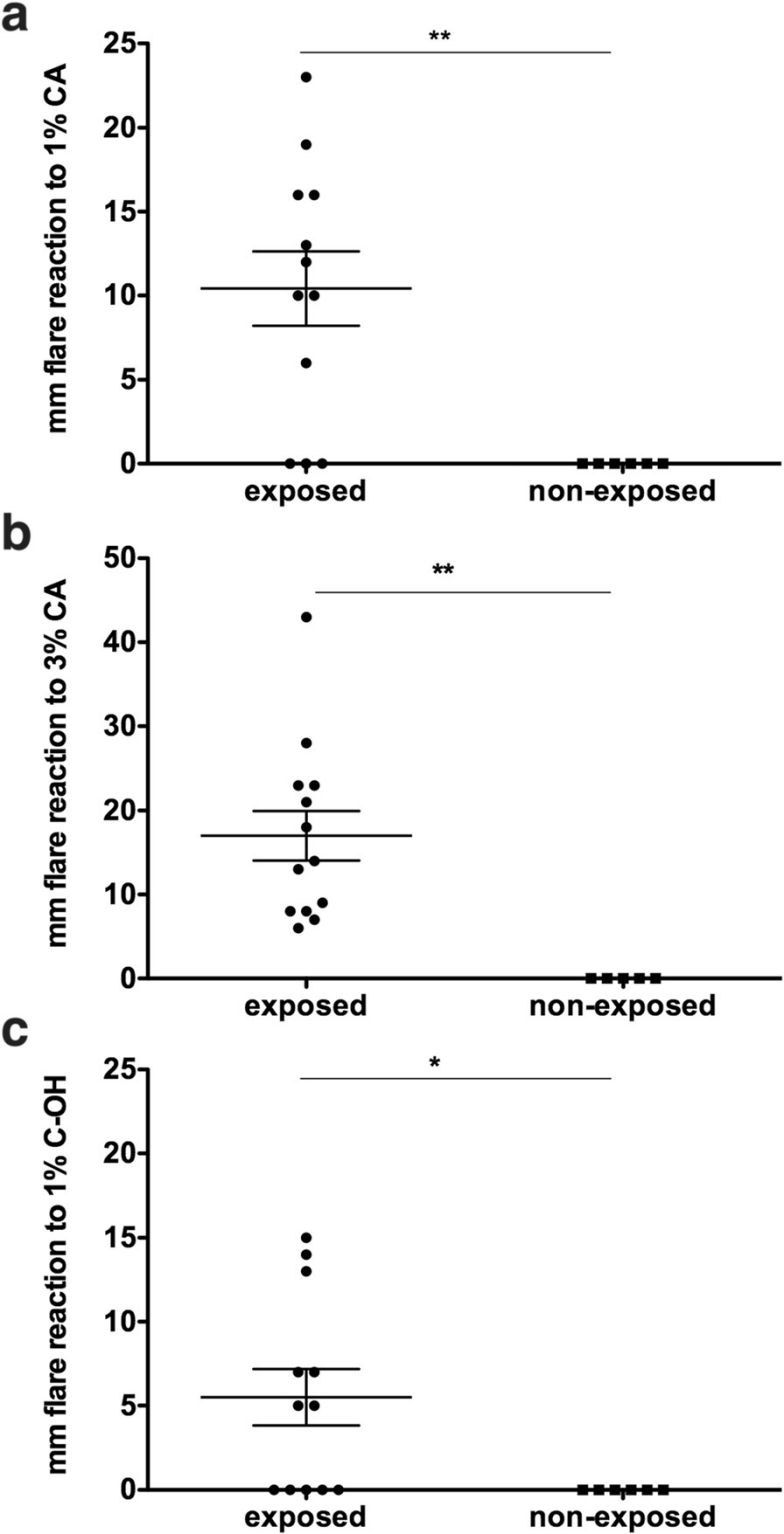
Skin prick test analysis. Wheal and flare reactions (mm) of 17 healthy volunteers with self-reported exposition to disco smoke or shisha and the patient (*n* = 12) or volunteers without exposition (*n* = 6) are depicted with **a** 3 % cinnamon aldehyde in 0.9%NaCl, **b** 1 % cinnamon aldehyde in Vaseline®, and **c** 1 % cinnamon alcohol in Vaseline®. Groups were compared using Mann-Whitney U test. * *p* < 0.05, ** *p* < 0.01

Volunteers were interviewed and divided into one group with previously self-reported exposure to disco fog or shisha, and one non-exposed group. Most of the tested volunteers were females. The mean age of exposed persons ranged 4.5 years higher than of non-exposed. The size of positive type I skin reactions significantly correlated with a history of exposure (Fig. 2).

## Methods

### Patient and volunteers

#### Diagnosis of patient with severe adverse reaction

After the second severe adverse reactions to spiced food the patient could be diagnosed in the Allergy clinic of the Medical University Vienna using standard skin prick series, as well as in prick-to-prick testing on the back 16 substances that were suspected by the patient, some of them also after boiling them 5 min. The foods were 1: blueberry raw, 2: blueberry boiled, 3: raspberry raw, 4: raspberry boiled, 5: poppy seeds boiled, 6: mango raw, 7: mango boiled, 8: papaya raw, 9: papaya boiled, 10: physalis raw, 11: potato raw, 12: tomato raw, 13: Nutella, 14: orange, 15: cinnamon powder in 0.9 % NaCl, 16: ficus.

#### Skin testing in healthy volunteers

The study was conducted in accordance with the Helsinki Declaration and approved by the local ethics committee of the Medical University Vienna. 17 volunteers were recruited and interviewed using a questionnaire for pre-existent allergies or atopy, fragrance hypersensitivity, exposure to smoke from dance floor machines, or to shisha. 2 groups were defined, 1.) Exposed to fog or shisha (*n* = 10), mean age 32.8 (+/- 10.8), 3 male/7 females, and 2.) Non-exposed (*n* = 8), mean age 37,3 (+/- 13.0), 1 male/7 females. All volunteers were pricked on the volar forearms with: 3 % cinnamon aldehyde (1: in 0,9 % NaCl; 2: in glycerol, 3: in glycerol boiled, 4: in smoke fluid Eurolite®, 5: in smoke fluid Eurolite® boiled, or 6: 1 % cinnamon aldehyde in Vaseline®, or 7: cinnamon alcohol in Vaseline®), and the size and intensities of skin reactions evaluated and photographed after 20 min.

#### Sampling and chemical analysis of fog

A fog machine (Profog K-120, Hildesheim, Germany) was purchased and operated with fog fluid (Eurolite® E) alone, followed by fog fluid supplemented with cola fragrance (Eurolite®) (0.4 ml/L) as prescribed by the manufacturer. The fogs were collected by solid phase micro extraction based sampling devices (SPME Portable Field Sampler, 1 cm fiber length coating 75 μm Carboxen/PDMS, Sigma Aldrich, Germany) during 15 and 60 min of operation. The samplers were conditioned according to the recommendation of the manufacturer and stored in pre-cleaned stainless steel tubes with a Polytetrafluoroethylene (PTFE) lined screw cap. After sampling they were put back to the storage devices and sent to the laboratory for gas chromatography-mass spectrometry (GC-MS) analysis.

The volatile fraction was desorbed directly in the hot injector of a GC-MS system (Agilent 6890 GC with 5973 mass selective detector equipped with a CTC combi PAL automatic injector) at 270 °C. Separation was done on a HP-5MS column (30 m*0.25 mm i.d., 1 μm fim thickness) with Helium as the carrier gas at 30 cm/s linear velocity in the constant flow mode. A temperature programme starting from -10 °C (1 min) with a ramp of 12 °C/min to 280 °C was used. The mass spectrometer was operated in the scan mode with a scan range of 20–300 amu with 3.2 scans per second.

#### Statistical analyses

Statistical analyses were conducted using Mann-Whitney test with GraphPad Prism 5 software (GraphPad, San Diego, CA, USA); *p* < 0.05 was considered statistically significant.

## Discussion

We propose that fog machines during operation may complex glycerol fluids with added fragrances and thereby transform them into complete immunogens. The repeated exposure to these aerosols mimics the prime and boost principle of specific respiratory sensitization, a mechanism that is exploited in numerous mouse models of allergy and asthma [27]. It is further well known that respiratory allergy may be causative for food allergy.[28] Starting from a case we illustrate for the first time i) that repeated aerosol exposure to a fragrance, cinnamon aldehyde, may cause specific hypersensitivity, and ii) that consequently, ingestion of the same flavor in food may elicit a severe immediate type reaction as the first symptom. In light of the current literature we propose that in the series of events the crossreactivity in this patient may have expanded from closely chemically related substances cinnamon aldehyde/acid/alcohol [2] to other benzaldehyde derivatives p-cymene and 4-hydroxy-benzyl-isothiocyanat, as illustrated in Additional file 1: Figure S1. In contact allergy of adults and children crossreactivities among fragrances are well known and clinically considered [29, 30]. In type I allergy potential crossreactivities to chemicals are less investigated and understood, but fragrances are also able to release histamine in fragrance hypersensitive patients [31], even though an IgE-mediated mechanism could not be confirmed [32].

We propose that case evaluations of patients with severe food reactions, especially to spices [33], should therefore comprise the question of exposure to artificial fogs.

To further support our findings we performed a screening study in healthy volunteers, using cinnamon aldehyde in various formulations for skin prick testing (Fig. 2). We decided for prick testing as opposed to patch testing based on the fact that also patch test for food allergy may show a variety of 0–100 % in terms of sensitivity, specificity, and predictive values [34]. Knowing the operation mechanism of fog machines based on glycerol-containing fluids and fragrances to camouflage the acrolein smell, it seemed plausible that also in shisha similar effects could be expected: Tobacco leaves, being soaked in glycerin for elasticity, render acrolein during smoking, therefore fragrances are added. Indeed, the skin test clearly identified persons with a history of exposure to fogs, especially from shisha pipes. The group of exposed persons was slightly younger than the non-exposed, skin test-negative persons. Among the exposed persons were besides the patient also three family members of the patient living in the same household. Albeit their fog machine exposure had been less intense, two of them had recently developed intolerance against cinnamon containing cookies, in the form of reflux 30 min after consumption.

Our approach to mimic the generation of complete allergens by the operation of a fog machine led to the identification of cinnamon aldehyde in the collected fogs when the fragrance “cola” had been added. Besides, higher glycerol derivatives such as 1,3,5-trioxane, 1,2-propanediol, di- and triethylene glycol were found. Against our expectations acrolein could not be detected, likely due to its high degree of instability and chemical reactivity [35]. Acrolein is a well known irritant and carcinogen and can be detected in aerosols and liquid extracts from tobacco smoke or E-cigarettes [36], and is used for biomonitoring smoke exposure [37].

## Conclusions

We anticipate that during heating of glycerol containing fluids in fog machines or shisha acrolein is formed with the potential for complexing added cinnamon fragrances. Moreover, also higher glycerol compounds may associate physically with cinnamon aldehyde to form antigenic particles, complete antigens, in the smoke

Overall, based on our data we propose that the addition of fragrances into fog machines and into shisha may lead to specific immediate type hypersensitivity.

### Consent

Written consent to publish was obtained from the patient.

## Additional file

Additional file 1: Figure S1. A) Chemical formulas of substances related to cinnamaldehyde which were related to adverse reactions in the patient; B) Chemical formulas of substances contained in smoke fluids; C) Glycerol can be transformed to acrolein under subtraction of water, and *vice versa*. (TIF 158 kb)
