# Supplementary figures and images for: Prime and boost aerosol exposure via fog machine or shisha smoke followed by cinnamon hypersensitivity and anaphylaxis to spiced food
Source: World Allergy Organ J. 2016 Jan 27;9:4. doi: 10.1186/s40413-016-0091-6 (PMC4730641; doi:10.1186/s40413-016-0091-6)

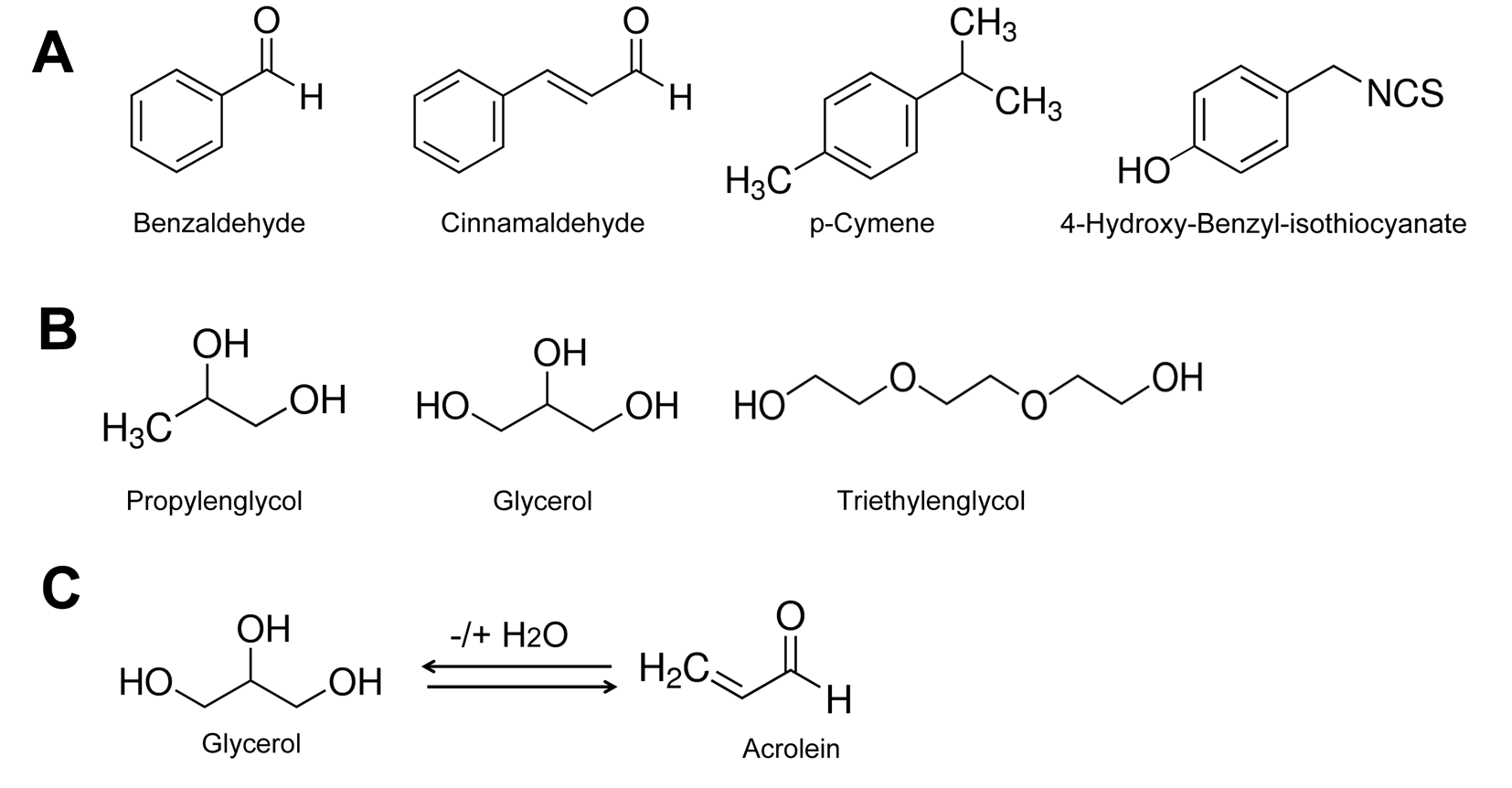

Supplement: Additional file 1: Figure S1. — A) Chemical formulas of substances related to cinnamaldehyde which were related to adverse reactions in the patient; B) Chemical formulas of substances contained in smoke fluids; C) Glycerol can be transformed to acrolein under subtraction of water, and vice versa. (TIF 158 kb) [file 40413_2016_91_MOESM1_ESM.tif]
